# Primary Burkitt lymphoma of the supraglottic larynx: a case report and review of the literature

**DOI:** 10.1186/s13256-017-1209-3

**Published:** 2017-03-10

**Authors:** Alexandra E. Quimby, Lisa Caulley, Danielle Rodin, Bibianna Purgina, Libni Eapen, Luke Shier, Stephanie Johnson-Obaseki

**Affiliations:** 1 Department of Otolaryngology – Head and Neck Surgery, University of Ottawa, 501 Smyth Rd, Ottawa, ON K1H 8L6 Canada; 2 Department of Radiation Oncology, University of Toronto, 150 College Street, FitzGerald Building, Rm 106, Toronto, ON M5S 3E2 Canada; 3 Department of Pathology and Laboratory Medicine, University of Ottawa, 451 Smyth Rd, Rm 4155, Ottawa, ON K1H 8M5 Canada; 4 Department of Radiation Oncology, University of Ottawa, 501 Smyth Rd, Ottawa, ON K1H 8L6 Canada

**Keywords:** Burkitt, Lymphoma, Larynx, Supraglottis, Irradiation, Case report

## Abstract

**Background:**

Burkitt lymphoma is a high-grade B cell lymphoma which accounts for less than 1% of all adult cases of non-Hodgkin lymphoma. Rare instances of Burkitt lymphoma developing secondary to prior irradiation have been described in the literature.

**Case presentation:**

We report a case of a 90-year-old white woman with a recent history of irradiation for Hodgkin lymphoma, who presented with primary Burkitt lymphoma of the supraglottic larynx. She underwent emergency awake tracheostomy with biopsy. A histopathological examination confirmed non-Hodgkin, B cell lymphoma of Burkitt type. Given her age and poor functional status, she underwent treatment with palliative radiotherapy.

**Conclusions:**

A literature review was performed to clarify the clinical characteristics of radiation-induced Burkitt lymphoma in the head and neck, as well as its diagnosis and management. The present case represents the second case of radiation-induced Burkitt lymphoma in the head and neck in the reported literature, and the first in the supraglottic larynx. It highlights the need to maintain a broad differential in the assessment of malignancies of the larynx, particularly in patients with a prior history of radiation treatment.

## Background

Burkitt lymphoma is a high-grade B cell lymphoma that was first described by Denis Burkitt in the mid-twentieth century as an aggressive malignancy of the mandible in African children [1]. The World Health Organization (WHO) classifies Burkitt lymphoma as endemic, sporadic, or immunodeficiency-associated, based on underlying mechanisms of cancer formation. Endemic Burkitt lymphoma is that which occurs in African children, and is almost always associated with Epstein–Barr virus (EBV) infection. Sporadic Burkitt lymphoma refers to cases occurring worldwide with no specific geographic distribution, and accounts for 1 to 2% of adult lymphoma cases in the USA and Western Europe [2–4]. Tumor cells are EBV-positive in only 15 to 30% of cases of sporadic Burkitt lymphoma occurring in the USA [5]. Sporadic Burkitt lymphoma primarily involves lymph nodes in the abdomen, although occasionally occurs extranodally in the ovaries and kidneys and less frequently presents in the head and neck as cervical adenopathy. Extranodal head and neck presentations account for less than 10% of all cases of sporadic Burkitt lymphoma [6]. Rarely, Burkitt lymphoma develops secondary to prior irradiation, with only a few previous cases having been reported in the literature [7].

We present the case of a 90-year-old woman who was diagnosed with Burkitt lymphoma of the supraglottic larynx following radiation for a previous Hodgkin lymphoma. This is a rare extranodal head and neck presentation of Burkitt lymphoma, particularly in light of the patient’s advanced age and her previous radiation treatment for Hodgkin lymphoma.

## Case presentation

**Fig. 1:**
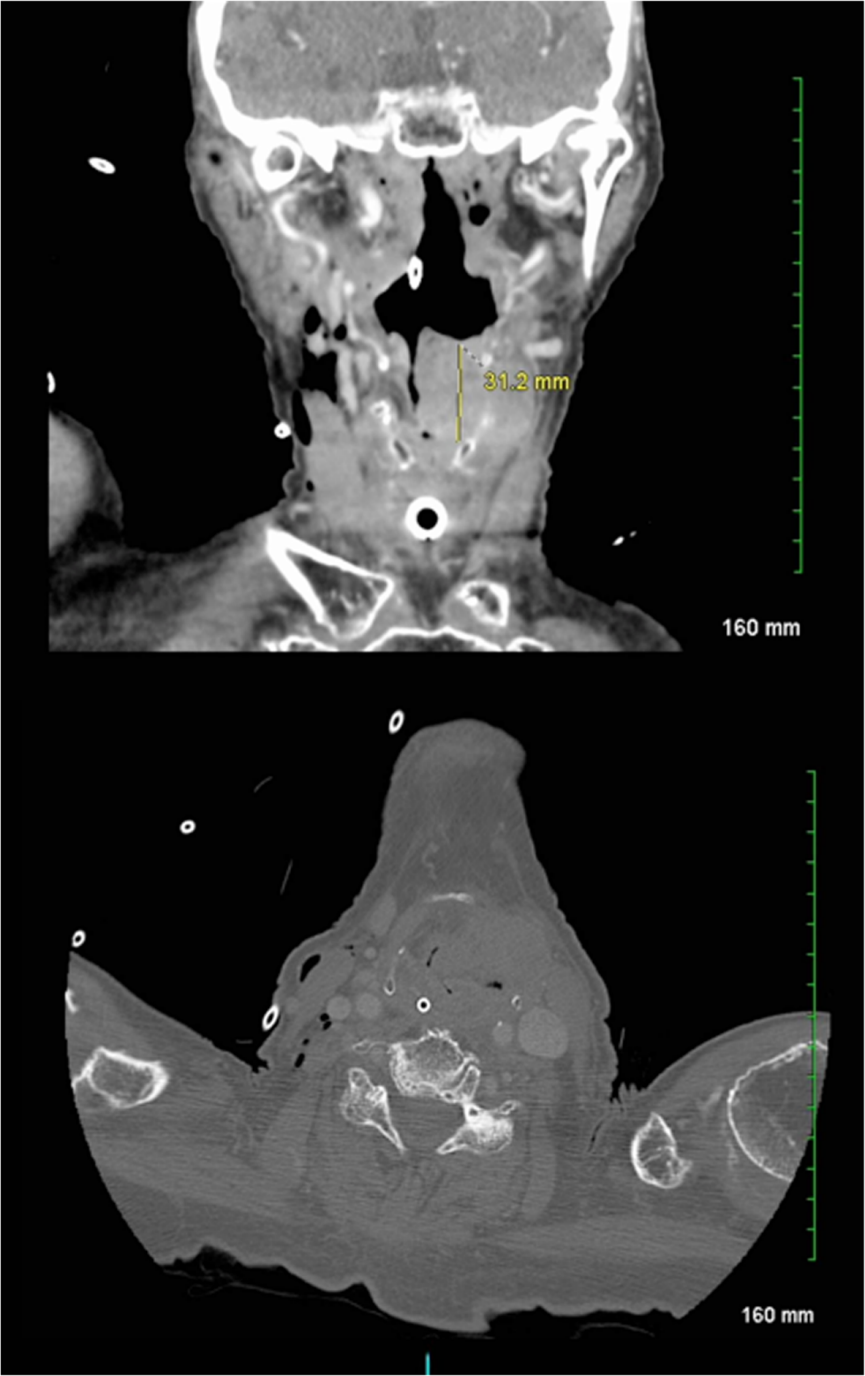
Initial computed tomography scan of the patient’s neck showing left neck mass involving the left supraglottic larynx and extending to the glottis and paraglottic space. The mass extended out through the thyrohyoid membrane and involved the left strap muscle

A 90-year-old white woman presented with a large neck mass and a 6-week history of increasing dysphonia, stridor, and left-sided otalgia. On examination, there was a firm 2×3 cm left-sided level III neck mass with no overlying skin changes. Flexible fiberoptic nasolaryngoscopy revealed a large exophytic mass with complete airway obstruction. A computed tomography (CT) scan revealed the mass to measure approximately 3.3×3.7×3.1 cm. It was shown to involve her left supraglottic larynx and extend down to at least the level of her glottis (Fig. 1).

Eight years earlier, she was diagnosed as having primary Hodgkin lymphoma in a left cervical node. Pathology of the node revealed large atypical cells, Reed–Sternberg cells, and a fine reticular sclerosis pattern. Immunophenotyping performed on a paraffin block demonstrated positive staining of the large atypical cells with cluster of differentiation (CD) 15 and CD30. The stains were negative for leukocyte common antigen (LCA), CD20, EBV-encoded small ribonucleic acid (RNA) (EBER). She was classified as Stage 1A classical Hodgkin lymphoma, mixed cellularity type, which was a rare diagnosis at age 82. Due to her localized disease, she was treated with definitive radiotherapy at a dose of 3500 cGy in 20 fractions delivered over 4 weeks.

Her past medical history was otherwise significant for a left hip fracture and surgical repair 1 year earlier, as well as mild cognitive decline, with difficulties in sequencing, planning, memory, attention, and insight. Due to her cognitive status, she was reliant on family members for many of her daily activities.

**Fig. 2:**
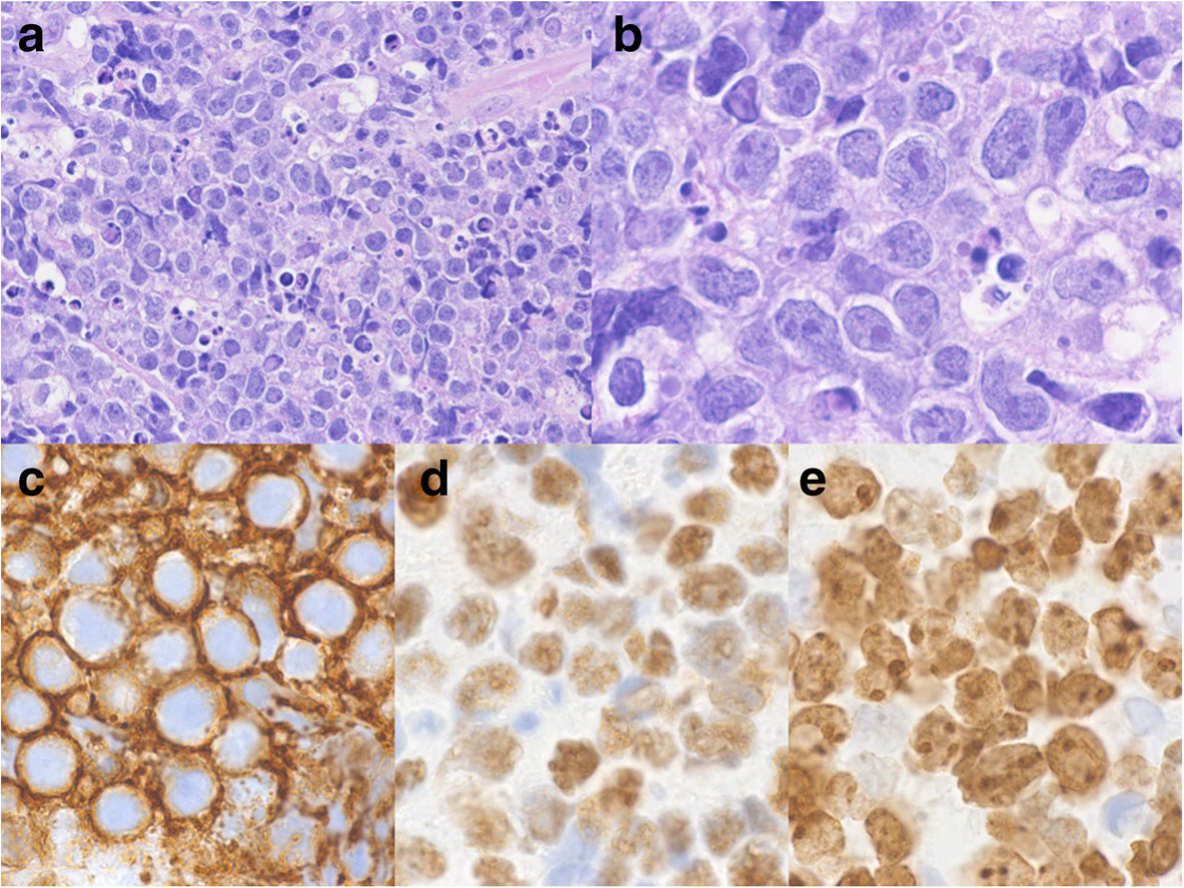
Supraglottic larynx biopsy demonstrating infiltrating sheets of poorly differentiated lymphoid cells, numerous mitoses, and tingible body macrophages imparting a “starry-sky” pattern. **a** Hematoxylin and eosin (H&E) stain 40× magnification. **b** H&E stain, 100× magnification. **c** Tumor cell CD20 reactivity, 100× magnification. **d** Tumor cell c-myc reactivity, 100× magnification. **e** Tumor cell Ki-67 reactivity

Following presentation, she underwent an emergency awake tracheostomy with laryngoscopy and biopsy of the supraglottic tumor. Microscopic examination revealed infiltrating sheets of poorly differentiated lymphoid cells with round nuclei containing several small nucleoli and dispersed chromatin and a small amount of amphophilic cytoplasm (Fig. 2). A high mitotic rate and a “starry sky” appearance, due to numerous tingible body macrophages, were noted. An extensive immunohistochemical panel was performed and the tumor cells were immunoreactive for CD20, BCL6, PAX 5, c-myc, and EBER (Fig. 2). Ki-67, a proliferation index marker, highlighted almost 100% of cells (Fig. 2). BCL2 was negative. Fluorescence *in situ* hybridization was performed and demonstrated the confirmatory *myc* rearrangement. The final histopathological diagnosis was a non-Hodgkin, B cell lymphoma of Burkitt type. This was distinct from her previous diagnosis of Hodgkin lymphoma 8 years prior.

This case was reviewed at a multidisciplinary tumor board to determine the appropriate course of management. She was not deemed a candidate for systemic therapy in light of her age and functional status. She underwent a 7-day course of high-dose prednisone (100 mg daily) before starting palliative radiation at a dose of 3600 cGy in 18 fractions. She subsequently developed mucositis, and was treated palliatively for symptom control. She had recurrent aspiration and developed bacteremia secondary to aspiration pneumonia for which she opted to forgo antibiotics. She died approximately 3 months following her initial presentation, before a repeat laryngeal examination could be conducted.

## Discussion

Squamous cell carcinoma is the most frequently encountered cancer of the larynx; less commonly encountered laryngeal cancers include adenocarcinoma, adenoid cystic carcinoma, and neuroendocrine tumors. Malignant lymphomas account for less than 1% of all laryngeal tumors [8]. While the head and neck is the second most common extranodal site of involvement in non-Hodgkin lymphoma (NHL), the larynx is rarely involved, with structures typically affected being Waldeyer’s ring, ocular adnexal structures, the nasal cavity, nasopharynx, paranasal sinuses, thyroid gland, as well as salivary glands [9, 10]. Among cases of NHL affecting the larynx, the most common classifications described in the literature include mucosa-associated lymphoid tissue (MALT) lymphoma, plasmacytoma, and diffuse large B cell lymphoma. The supraglottic larynx is most commonly involved [9]. Among extranodal head and neck presentations of sporadic Burkitt lymphoma, the nasopharynx and tonsils were the most common sites of occurrence in one large case series of American sporadic Burkitt lymphoma [3]. Other extranodal sites of involvement within the head and neck, which occur in less than 5% of patients with sporadic Burkitt lymphoma, include the nasopharynx, tonsils, palate, sinuses, nasal septum, and other facial bones [3, 6, 11, 12]. This is the first reported case of primary Burkitt lymphoma of the larynx.

The aggressiveness of primary treatment for Hodgkin lymphoma, especially radiation therapy, is a primary risk factor in the development of subsequent lymphoma [13]. Our patient’s development of non-Hodgkin, Burkitt-type lymphoma following recent irradiation for cervical Hodgkin lymphoma is also an exceedingly uncommon occurrence. NHL is reported to occur following treatment for Hodgkin lymphoma at a rate of only 0.9% at 6.7 years, and 1.6% at 15 years [14].

To ascertain the frequency of radiation-induced Burkitt lymphoma, we performed a detailed electronic search of PubMed, MEDLINE, and Embase for studies reporting on radiation-induced Burkitt lymphoma. The following MeSH terms were used in varying combinations: *Burkitt lymphoma*, *radiation-induced*, *radiotherapy*, *radiation-injury*, and *neoplasm*. The search identified 22 potential studies, of which one was relevant for the qualitative synthesis [15]. We subsequently performed a hand-search of the literature which yielded one additional study for inclusion in our analysis [7]. These two studies together identified a total of 18 cases of Burkitt lymphoma which developed following prior radiation treatment for primary malignant neoplasm (Table 1). One case occurred in the mandible of a patient previously treated for oropharyngeal cancer [15]. This is, to the best of our knowledge, the only previously described case of Burkitt lymphoma developing in the head and neck secondary to prior radiation treatment.

**Table 1:** Reported cases of Burkitt lymphoma occurring following prior radiation treatment for primary malignant neoplasm

Case	Patient age^a^/sex	Primary neoplasm	Treatment of primary neoplasm	Time from primary neoplasm to Burkitt lymphoma
1	13/M	Hodgkin’s lymphoma	Chemo + RT	132 months
2	21/F	Hodgkin’s lymphoma	Chemo + RT	90 months
3	45/M	Hodgkin’s lymphoma	Chemo + RT	84 months
4	34/M	Hodgkin’s lymphoma	Chemo + RT	36 months
5	19/F	Hodgkin’s lymphoma	Chemo + RT	168 months
6	39/M	Hodgkin’s lymphoma	Chemo + RT	78 months
7	67/M	Hodgkin’s lymphoma	Chemo + RT	28 months
8	32/M	Hodgkin’s lymphoma	Chemo + RT	97 months
9	38/M	Hodgkin’s lymphoma	Chemo + RT	120 months
10	20/M	Hodgkin’s lymphoma	Chemo + RT	100 months
11	25/M	Hodgkin’s lymphoma	Chemo + RT	99 months
12	36/M	Hodgkin’s lymphoma	Chemo + RT	65 months
13	44/F	Hodgkin’s lymphoma	Chemo + RT	102 months
14	33/M	Hodgkin’s lymphoma	Chemo + RT	112 months
15	21/M	Hodgkin’s lymphoma	RT	72 months
16	39/F	Hodgkin’s lymphoma	Chemo + RT	88 months
17	13/F	Rhabdomyosarcoma	Chemo + RT	6 years
18	71/M	Oropharyngeal CA	RT	15 years

Cases 1–16 from Salloum *et al*., 1996 [7]; cases 17 and 18 from Nagasaki *et al*., 2009 [15]

*CA* cancer, *Chemo* chemotherapy, *F* female, *M* male, *RT* radiotherapy

^a^Patient age at time of diagnosis of Burkitt lymphoma

The diagnosis of Burkitt lymphoma is made on the basis of histology and immunohistochemistry. On histologic examination, it is characterized by neoplastic lymphoid cells with round nuclei, dispersed chromatin, several small nucleoli, and a small amount of amphophilic cytoplasm. Mitoses are numerous and the tumor cells are admixed with tingible body macrophages, which are macrophages phagocytosing apoptotic debris, imparting a “starry sky” pattern. Most Burkitt lymphomas are immunoreactive for surface immunoglobulin M (IgM), as well as pan-B cell antigens including CD19, CD20, and CD22, and co-express CD10, CD43, and BCL6, but not CD5, CD23, BCL2, or TdT [16, 17] Nuclear staining with c-myc antibody is positive: in all cases of Burkitt lymphoma, a translocation exists between the *c-Myc* gene and either the *IgH* gene, as is found in 80% of cases, or either the kappa or lambda light chain gene, found in the other 20% [18]. Another important feature of Burkitt lymphoma is that nearly 100% of the tumor cells are positive for Ki-67, a proliferation index marker, in keeping with the tumor’s extremely rapid rate of growth: the doubling time of Burkitt lymphoma is among the fastest of any human malignancy [19]. Of note, EBV antibody titers are found to be positive in almost 100% of patients with endemic Burkitt lymphoma, but are only positive in 15 to 30% of patients with sporadic Burkitt lymphoma [16].

Given its fast-growing nature, prompt diagnosis of Burkitt lymphoma is essential to prevent local growth with compression of vital structures in the head and neck, or early widespread dissemination. The former occurred in the case we described, resulting in airway obstruction. Typical staging consists of abdominal and chest CT, a bone marrow biopsy, and cerebrospinal fluid (CSF) testing. Due to our patient’s advanced age, poor functional status, and palliative treatment approach, a staging workup was not performed. Although localized early stage Burkitt lymphoma is highly responsive to chemotherapy, advanced age and high tumor burden at presentation are poor prognostic factors [16, 20].

The treatment of Burkitt lymphoma typically consists of intermittent high-dose chemotherapy, most commonly using cyclophosphamide, vincristine, doxorubicin, and methotrexate. Rituximab, a monoclonal CD20 antibody, has also shown some promise when used in conjunction with a typical chemotherapeutic regimen [21]. Tumor debulking may be beneficial as an adjunct therapy when the anatomical location is permissive, although its role in the treatment of head and neck presentations of Burkitt has not been well studied [22, 23]. When prognosis is poor, as in the case described, locoregional radiation plays a palliative role in providing local symptom control [24].

## Conclusions

Burkitt lymphoma is a B cell NHL with an extremely fast rate of growth, necessitating prompt diagnosis. Sporadic Burkitt lymphoma occasionally presents extranodally in the head and neck, although the most common head and neck presentation is cervical adenopathy. Rarely, radiation therapy has been linked to the subsequent development of Burkitt lymphoma. This case report describes a rare presentation of Burkitt lymphoma of the larynx in a 90-year-old patient previously irradiated for Hodgkin lymphoma. It represents the first reported case of Burkitt lymphoma in the larynx, and only the second described case of irradiation-induced Burkitt lymphoma occurring in the head and neck. It highlights the need to maintain a broad differential in the assessment of malignancies of the larynx, particularly in the context of a prior history of radiation treatment. Biopsy is a necessary step in the diagnosis of Burkitt lymphoma and in subsequent treatment planning, including selection of an appropriate chemotherapeutic regimen. When detected early, Burkitt lymphoma has a favorable prognosis and high remission rates. However, high tumor burden, advanced age, and poor functional status are associated with disease progression. In advanced stage or chemoresistant disease, there is good evidence that radiotherapy provides local symptom control.

## Abbreviations

CD: Cluster of differentiation
CSF: Cerebrospinal fluid
CT: Computed tomography
EBER: Epstein–Barr virus-encoded small RNA
EBV: Epstein–Barr virus
LCA: Leukocyte common antigen
MALT: Mucosa-associated lymphoid tissue
NHL: Non-Hodgkin lymphoma
WHO: World Health Organization
